# Moderating Effects of Loneliness on COVID-19-Related Stress and Anxiety in Middle and Later Life in South Korea

**DOI:** 10.1093/geroni/igab046.539

**Published:** 2021-12-17

**Authors:** Sukyung Yoon, Soo Chan Choi

**Affiliations:** 1 University of Wyoming, Laramie, Wyoming, United States; 2 Yonsei University, Seoul, Seoul-t'ukpyolsi, Republic of Korea

## Abstract

Many people have suffered from psychological distress in the form of stress, loneliness, and anxiety resulting from the COVID-19 epidemic (Havnen et al., 2020; Luchetti et al., 2020). Along with these factors, physical health (hereafter health), resilience, and living arrangements as protective factors were examined. The research aims were to investigate 1) factors affecting the association between COVID-19-related stress (hereafter stress) and anxiety, and 2) moderating effects of loneliness on this association. Data was collected on 450 middle-aged and older adults (ages 45 through 76) living in South Korea during COVID-19. A multi-group path analysis was employed. Measurement invariance was examined by comparing unconstrained and fully constrained models. Both models fit. Moderating effects of loneliness existed. Stress was negatively associated with health and living arrangements for people with both higher and lower levels of loneliness. Health was positively associated with resilience for both groups. Resilience was negatively associated with anxiety for both groups. For people with higher levels of loneliness only, stress and health were negatively associated with resilience and anxiety, respectively. The association between stress and anxiety was significant for both groups. However, the impact of stress on anxiety was significantly larger for people with higher levels of loneliness than for people with lower levels of loneliness. Health practitioners and service providers should develop programs to maintain and promote resilience, social support, and good health among middle-aged and older adults in South Korea to mitigate negative mental health consequences during the pandemic.

